# Effects and safety of intranasal phototherapy for allergic rhinitis: Study protocol for a single-center, randomized, parallel (acupuncture-controlled), open-label, investigator-initiated, pilot study

**DOI:** 10.1097/MD.0000000000021183

**Published:** 2020-07-24

**Authors:** Jeongin Kang, Goeun Lee, Miju Son, Youngeun Kim, Namhun Heo, Donghyo Lee

**Affiliations:** a Department of Ophthalmology, Otolaryngology, and Dermatology, College of Korean Medicine, Woo-Suk University, Jeonju; b Department of Oriental Rehabilitation, National Rehabilitation Center, Seoul; c Clinical Medicine Division, Korea Institute of Oriental Medicine; d Future Medicine Division, Korea Institute of Oriental Medicine, Daejeon; e Clinical Trial Center, Soonchunhyang University Hospital, Cheonan, Korea.

**Keywords:** allergic rhinitis, intranasal phototherapy, acupuncture, randomized controlled trial, study protocol

## Abstract

**Introduction:**

Allergic rhinitis (AR) is an immunoglobulin E (Ig E)-mediated inflammatory disease. Intranasal phototherapy is a promising treatment modality because it has a profound immunosuppressive effect, but the evidence of its use for AR is insufficient. Therefore, rigorously designed randomized controlled trials (RCTs) are needed. Our objective is to describe the protocol for an RCT to assess the effects and safety of intranasal phototherapy for the treatment of AR.

**Methods and analysis:**

This is a study protocol for a single-center, randomized, parallel (acupuncture-controlled), open-label, investigator-initiated, pilot study. A total of 80 patients with AR will be randomly assigned to the intranasal phototherapy or acupuncture group at a 1:1 ratio. The participants will receive intranasal phototherapy with medical or acupuncture treatment for 20 minutes, 3 times a week for 4 weeks. The primary outcome will be the mean change in the total nasal symptom score (TNSS) from baseline to 4 weeks. The secondary outcomes will include the Rhinoconjunctivitis Quality of Life Questionnaire (RQLQ) score, Nasal Endoscopy Index, total serum immunoglobulin E (Ig E) level and eosinophil count.

**Discussion:**

The findings of this study will provide the basis for the design and implementation of RCTs investigating the effects and safety of intranasal phototherapy for AR. Additionally, it will provide preliminary evidence of intranasal phototherapy for use in AR.

**Trial registration:**

This study was registered at the Korean National Clinical Trial Registry, Clinical Research Information Service (KCT0004079).

## 1 Introduction

Allergic rhinitis (AR) is an inflammatory disease of the nasal membranes that results from an IgE-mediated immune reaction to allergen exposure.^[1]^ The major symptoms of AR are nasal congestion, rhinorrhea, nasal itching and sneezing, and it is reported that patients with AR often have poor quality of life (QOL). Moreover, AR disrupts sleeping, interrupts learning and reduces productivity at work.^[2]^

AR is a common respiratory disease that affects 10% to 25% of the global population,^[3]^ and it affects 22% to 29.2% of the pediatric population and 20% of the adolescent population in Korea.^[4]^ It has been reported that the prevalence of persistent allergic rhinitis (PAR) has increased over the last decade.^[5]^

The management of AR involves allergen avoidance and medication such as antihistamines and nasal decongestants.^[6]^ Antihistamines relieve the symptoms of AR, but symptom relief may be incomplete,^[7]^ and long-term use of nasal decongestants can cause rhinitis medicamentosa.^[8]^ Additionally, there are some patients whose symptoms do not improve completely even with these medications and who have some limitations and special considerations in terms of using these medications, such as patients who are pregnant or breastfeeding.^[9]^

Low-level laser therapy (LLLT) is a promising treatment modality in Korean medical fields, and intranasal phototherapy represents an alternative intervention in the treatment of AR.^[10,11]^ Intranasal phototherapy is the application of light into the nasal cavity to relieve the symptoms of AR by inducing apoptosis of immune cells to suppress the reaction of inflammatory mediators^[12]^ and is expected to relieve the symptoms of AR for patients who do not respond well to conventional therapy.^[13]^ However, further studies are required on this topic to draw reliable conclusions about the effects and safety.^[14]^

We designed a single-center, randomized, parallel, open-label, investigator-initiated, pilot study to validate the clinical effects and safety of intranasal phototherapy for AR compared to those of acupuncture treatment, which is used as a usual care. The results of this study are expected to provide preliminary evidence of intranasal phototherapy for use in AR.

## 2 Methods

### 2.1 Objective

The aim of this study is to describe the protocol for a randomized controlled trial designed to clinically assess the effects and safety of intranasal phototherapy for AR compared with acupuncture treatment.

### 2.2 Study design and setting

This is a single-center, randomized, parallel (acupuncture-controlled), open-label, investigator-initiated, pilot study. This study will be conducted at the Woosuk University Korean Medicine Hospital, Jeonju, Republic of Korea.

**Figure 1:**
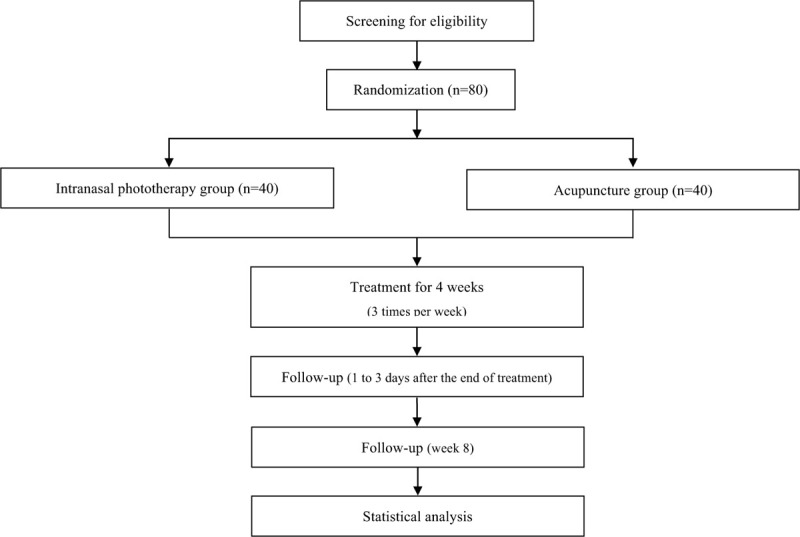
Flowchart of this study.

A total of 80 participants will be recruited for this trial and will be randomly allocated to 2 parallel groups: the intranasal phototherapy group or the acupuncture group. According to their group allocations, the participants will receive intranasal phototherapy or acupuncture treatment. The total study period will include a 4-week intervention and a 4-week follow-up. The study design is summarized in Fig. 1 and Table 1. The study protocol (version 1.1) was developed as required by the Standard Protocol Items: Recommendations for Interventional Trials (SPIRIT) guidelines (Additional file 1).

**Table 1:**
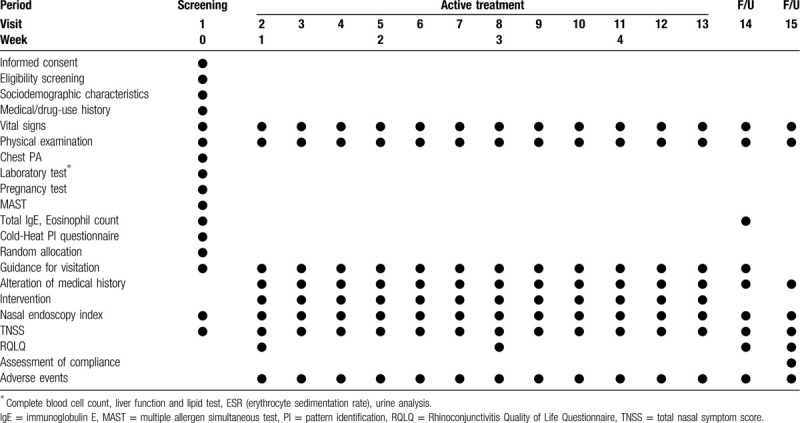
Study design schedule.

### 2.3 Recruitment

The recruitment posters will be displayed on the hospital bulletin board. The posters will contain brief descriptions and outline the purpose of this study, the inclusion criteria, and the intervention. A total of 80 participants will be recruited, and written informed consent will be obtained from all study participants before enrollment. All participants will have the right to participate or withdraw from the study at any time without penalty.

### 2.4 Participant inclusion and exclusion criteria

#### 2.4.1 Inclusion criteria

Participants will be included in this study if they meet all of the following criteria:

1. aged ≥19 years
2. presence of 2 or more symptoms of allergic rhinitis (nasal congestion, rhinorrhea, nasal itching and sneezing) and
3. positive reaction to an allergen in a multiple allergen simultaneous test (MAST).

#### 2.4.2 Exclusion criteria

Participants will be excluded if they meet any of the following conditions:

1. Currently taking any of the following medications or cannot stop taking medication that is unsuitable for this clinical trial. (However, enrollment in this trial is possible after the suggested minimum wash-out period before Visit2.)
  - Use of antihistamines/H1 blockers within the previous week
  - Use of corticosteroids (intranasal) within the previous 2 weeks
  - Use of corticosteroids (systemic) within the previous 30 days
  - Use of anticholinergic drugs within the previous week
  - Use of leukotriene receptor antagonists within the previous 4 weeks
  - Use of decongestants within the previous 3 days
  - Use of tricyclic antidepressants or phenothiazines within the previous 2 weeks
  - Use of nonsteroidal analgesics within the previous 2 weeks
  - Others (inappropriate medications judged by the investigator)
2. Received traditional Korean medicine treatments (herbal medicine, acupuncture, moxibustion, cupping, etc.) to treat allergic rhinitis within the previous 2 weeks
3. Received laser therapy to treat allergic rhinitis within the previous 2 weeks
4. Received treatments due to acute upper respiratory infections or sinusitis within the previous 4 weeks
5. Has nonallergic rhinitis such as vasomotor, infectious, drug-induced rhinitis, or other severe nasal illness
6. Has anatomical obstructions or deformities of the nasal cavity or underwent nasal surgery within the previous 6 months
7. Diagnosed with malignant neoplasm, anemia, active pulmonary tuberculosis, infection, or other severe systemic disease
8. Has a past history of active respiratory diseases such as asthma
9. Received immunotherapy or systemic corticosteroid therapy within the previous 5 years
10. Has hypersensitivity reactions to phototherapy or from taking related drugs
11. Has a scar on the irradiation (or acupuncture) spot
12. Are pregnant, planning a pregnancy or breastfeeding
13. Participated in other clinical trials within the previous 1 month
14. Has difficulties enrolling in this trial or receiving treatment
15. Others: ineligible for participation as judged by the investigator

### 2.5 Intervention

Subjects will be randomly assigned to 2 groups: an experimental group (medical device that emits low-level laser light) or a control group (acupuncture) at a ratio of 1:1. The experimental group will receive phototherapy in a single nasal cavity for 20 minutes, 3 times a week for 4 weeks. At each visit, the investigator decides on which side to receive phototherapy (right or left) based on the subjects symptoms and nasal endoscopy images. The medical device that will be used is the COBISTOP-S unit (Cngmedical Co., Ltd., Ulsan, Republic of Korea), which emits a low-level laser light consisting of a wavelength of 650 nm. The control group will receive acupuncture treatment for 20 minutes, 3 times a week for 4 weeks. Acupuncture will be performed by qualified Korean medicine doctors with at least 2 years of clinical experience. Ten acupuncture points (bilateral LI4, LI20, ST2 and ST36 and unilateral EX-1 and GV23) according to the “WHO Standard Acupuncture Point Location”^[15]^ were selected based on existing papers,^[16]^ and a 0.20 mm (diameter) × 30 mm (length) disposable needle (Dongbang Medical Co., Boryung-si, Republic of Korea) will be used. To induce de-Qi sensation, the needles will be manually manipulated 2 times, immediately after needling and before withdrawing. Acupuncture treatment details such as the acupuncture rationale, needling details, treatment regimen, cointervention, and practitioner background will conform to the Standards for Reporting Interventions in Controlled Trials of Acupuncture (STRICTA) checklist in Table 2.^[17]^

**Table 2:**
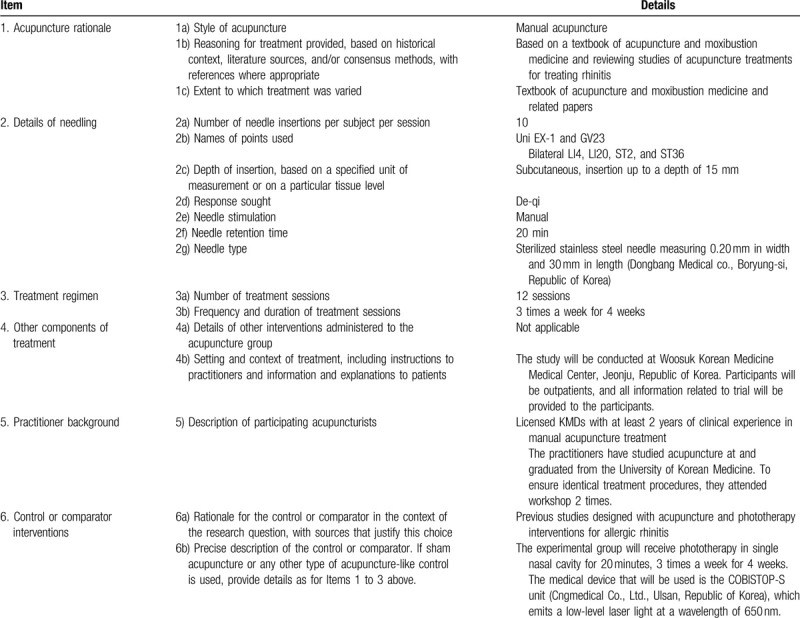
Details of acupuncture treatments for allergic rhinitis, based on the STRICTA 2010 checklist.

### 2.6 Outcome measures

#### 2.6.1 Primary outcome

The primary outcome is the change in the total nasal symptom score (TNSS) from baseline to 4 weeks. The TNSS assesses 4 nasal symptoms (nasal congestion, rhinorrhea, nasal itching and sneezing) on a five-point scale: 0, no symptoms; 1, mild; 2, moderate; 3, severe; and 4, very severe.^[18]^ The TNSS will be measured at every visit, and we will analyze the differences in effects between the 2 groups.

#### 2.6.2 Secondary outcomes

The secondary outcomes will include the change in the Rhinoconjunctivitis Quality of Life Questionnaire (RQLQ) score, Nasal Endoscopy Index, total serum immunoglobulin E (IgE) level, eosinophil count level, and adverse events. The RQLQ is a rhinoconjunctivitis-related quality of life instrument consisting of 28 items in 7 domains (activity limitation, sleep problems, nose symptoms, eye symptoms, non-nose/eye symptoms, practical problems and emotional function).^[19,20]^ The RQLQ score will be assessed 4 times: just prior to the start of the treatment, at visit 2 and visit 8, and at the 2 follow-up periods.

The Nasal Endoscopy Index assesses nasal cavity conditions according to 4 aspects: color (pale or hyperemia), dryness or dampness, rhinorrhea, and atrophy or edema.^[21]^ The Nasal Endoscopy Index will be evaluated at every visit. The instrument used will be the KAZAMA ENT treatment unit (KAU-3000 HARMONY, ENT Co., Ltd., Incheon, Republic of Korea).

We will also examine the total serum IgE level and eosinophil count at screening (visit 1) and visit 14 to evaluate the effects of intranasal phototherapy on allergy and inflammatory reactions.

#### 2.6.3 Other measures

To identify the correlation between the effects of the interventions and the cold-heat pattern, we will use the Cold-Heat Pattern Identification Questionnaire. The questionnaire consists of 15 items, which include 8 items related to cold and 7 items related to heat,^[22]^ and the questionnaire will be assessed at screening.

### 2.7 Randomization and allocation concealment

Participants who meet all the inclusion and exclusion criteria will receive a subject identification code. Accordingly, block randomization will be performed to assign the same number of subjects to the intranasal phototherapy or acupuncture group. A total of 80 participants will be randomly assigned to each group with a 1:1 ratio. A statistician who is not directly involved in this trial will generate a random allocation sequence using a computer program [Strategic Applications Software (SAS), version 9.3; SAS Institute Inc., Cary, NC]. The allocation for each randomization number will be put into individually sealed, consecutively numbered security envelopes. Consent will be obtained by a Korean medicine doctor, and before the random assignment, all participants will be informed that they will be assigned to 1 of 2 groups. After the participants have signed the informed consent form, the envelopes will be opened.

### 2.8 Blinding

The investigator performing the outcome assessments will be blinded to the group allocation data. However, the subjects and the investigators who perform the intervention will not be blinded due to the characteristics of the study. The subjects will receive intranasal phototherapy or acupuncture treatment according to their randomization number.

### 2.9 Sample size

Since the aim of this study is to determine preliminary feasibility, there will be no sample size calculation. We will conduct a pilot study with a sample size of 80 participants (40 in the intranasal phototherapy group and 40 in the acupuncture group) based on the previous pilot study,^[23]^ and we predict that the drop-out rate will be 20%. A total of 64 patients are predicted to complete the study. This sample size is considered to be sufficient to provide power analysis and sample size calculation for subsequent large-scale definitive RCTs.

### 2.10 Data management

The investigators will telephone the participants before every visit to promote participant adherence. If participants fail to attend treatment sessions, we will ask the reasons for the absence and encourage visits through telephone contact.

Regular monitoring will be conducted to control the quality of the data according to the planned protocol and standard operating procedures. Medipert, the clinical research organization (CRO) of this study, will send a clinical research associate (CRA) to Woosuk Korean Medicine Medical Center. The CRA will check whether the trial is conducted according to the approved protocol and whether the data are adequately recorded. Each participants contact or identifying information will be separately stored. Personal identity will not be disclosed. Additionally, investigators will not collect any other personal data. All data and records related to the clinical trials should be kept in a locked cabinet. Documents related to this clinical trial will be kept for 3 years after completion of the clinical trial.

### 2.11 Safety and adverse events

The participants will be educated to voluntarily report information about adverse events (AEs), and the investigator will check any expected or unexpected AEs. AEs such as nasal dryness, nasal septum perforation, burning, epistaxis (nosebleed), inflammation or stinging, or other AEs reported during the study period will be recorded in case report forms, and participant vital signs will be checked at every visit. The symptoms, date of occurrence and disappearance, severity, causal relationship with the treatment, other medications or treatments suspected to have caused the AE, and treatment for the AE will be recorded in detail. When serious AEs occur, the investigator will immediately report the event to the institutional review board (IRB) and decide whether to cease the treatment. Additionally, AEs associated with the intervention during the trial will be appropriately handled, and we will provide corresponding compensation according to insurance policy.

### 2.12 Statistical analysis

The statistical analysis, including of the primary and secondary outcomes, will be performed using the full analysis set based on the principle of intention-to-treat (ITT); the per-protocol (PP) analysis set will be used for the sensitivity analysis. We will apply the last-observation-carried-forward (LOCF) rule for missing data.

Baseline differences between groups will be assessed using an independent two-sample *t* test or the Wilcoxon rank-sum test for continuous variables and the Chi-Squared test or Fisher exact test for categorical variables. Paired *t* test or Wilcoxon signed-rank test will be performed to analyze differences in the primary and secondary outcomes before and after treatment in each group. To demonstrate noninferiority, we will check whether the upper limit of the one-sided 97.5% confidence interval (CI) of the difference in the primary outcome between the 2 groups is less than the noninferiority margin (2 points).^[16]^ A subgroup analysis will also be performed to explore differences in the effects of intranasal phototherapy or acupuncture treatment according to the cold-heat pattern, symptom severity, and so on.

All participants who received at least 1 session will undergo an adverse effect evaluation. All adverse effects reported during the clinical trial will be analyzed. To compare groups and the incidence frequency of adverse events related to the 2 groups, the Chi-Squared test or Fisher exact test will be used. The level of significance will be set at 0.05 (2-tailed), except for the primary outcome, and all analyses will be performed using SAS version 9.3 (SAS Institute. Inc., Cary, NC).

### 2.13 Ethics

The study will be conducted in compliance with the Declaration of Helsinki and the Korean Good Clinical Practice (KGCP) guidelines. This study protocol was approved by the Institutional Review Board of the Woosuk University Korean Medicine Hospital (WSOH IRB M1904-01-02) and registered in the Clinical Research Information Service (CRIS), which is a primary registry of the World Health Organization International Clinical Trials Registry (https://cris.nih.go.kr/cris/en/search/search_result_st01.jsp?seq=16811). Protocol modifications will be provided to the Institutional Review Board and the trial registry for their approval. Prior to participation, participants will be fully informed about the study and voluntarily sign an informed consent form.

The results of the study will be shared with health care professionals, traditional medicine associations, and other relevant organizations through conferences and will be published in peer-reviewed journals.

## 3 Discussion

The primary treatments currently used for AR are antihistamines, nasal decongestants, and nasal steroids. However, long-term use of these medications can cause side effects, and the effects of these medications do not last long once the medication is stopped.^[24]^ Due to the limitations of conventional therapies, there is a need to develop safe and effective treatments for AR.

Intranasal phototherapy has received growing interest as an alternative choice in the treatment of AR. In particular, intranasal phototherapy is expected to relieve the symptoms of AR for patients who cannot take conventional medications because of their contraindications or for those who do not respond well to conventional therapy. Phototherapy was initially limited to treating some localized skin lesions, but its scope is expanding as the clinical efficacy and mechanism have been revealed.^[25]^ The pathological mechanism of allergic disease is known to be affected by phototherapy. A recent study showed that LLLT significantly inhibited total IgE and IL-4 and improved histological damage to the epithelium in the nasal septum of ovalbumin-induced mice.^[26]^ Previous studies demonstrated that LLLT is an effective modality for treating AR, as it significantly alleviated rhinitis symptoms and improved QOL; additionally, these studies reported no serious side effects or complications.^[23,27,28]^ However, there are still limitations with the current research, including the differences in study design, laser power density, dosage, and wavelength. It is also categorized as a technology that requires further studies to validate its safety and effectiveness. The purpose of this pilot study is to clinically assess the effects and safety of intranasal phototherapy compared to usual care - acupuncture treatment - using a medical device that is already approved for the treatment of AR.

We designed a pilot study to validate the clinical effects and safety of intranasal phototherapy for AR compared to those of acupuncture treatment, which is used as a usual care. Previous studies have demonstrated that acupuncture is effective at reducing symptoms of AR and improving QOL.^[29–31]^ International, multicenter clinical study showed that 4 weeks of acupuncture treatment could alleviate the symptoms of PAR more effectively than sham acupuncture and waitlist groups.^[16]^ This result suggested that acupuncture could be an effective and safe treatment for AR, and we chose 10 acupoints and set the treatment period based on this study. Acupuncture has been used to treat AR in Korean medicine. It can avoid side effects that may occur during drug use and lower the days of drug use in patients with AR.^[18,32]^ Additionally, treating patients with AR in routine care with additional acupuncture treatment has a long-lasting therapeutic effect.^[31]^ Recently, studies on acupuncture for allergic diseases including AR have been increasing in the United States, Germany, and China.^[33]^ It is suggested that acupuncture treatment has a beneficial effect on AR.^[16,34]^ In Korea, National Health Insurance (NHI) has covered Korean medicine services including acupuncture, moxibustion, cupping, and herbal preparations.^[35]^

The committee for New Health Technology Assessment (nHTA) in Korea assessed phototherapy for AR and made conclusions that further studies are needed on this topic for its clinical application since there is insufficient research result on safety and effectiveness of the technology on AR patients.^[36]^ We expect that the results of this study will provide evidence to verify the effects of intranasal phototherapy and basis for the evaluation of nHTA.

There has been increased interest in the development of Clinical Practice Guideline (CPG) in Korean medicine for establishment of its evidence and Korean medicine related health insurance policy, and AR is one of the target diseases. We expect that this study results will contribute to the development of treatment interventions in Korean medicine and establish evidence for “intranasal phototherapy”, which is included as a key question in the Allergic Rhinitis Korean Medicine Clinical Practice Guideline.

## Author contributions

**Conceptualization:** Donghyo Lee.

**Data curation:** Jeongin Kang, Namhun Heo.

**Funding acquisition:** Donghyo Lee.

**Investigation:** Jeongin Kang, Donghyo Lee.

**Methodology:** Jeongin Kang, Goeun Lee, Miju Son, Youngeun Kim, Namhun Heo, Donghyo Lee.

**Writing – original draft:** Jeongin Kang.

**Writing – review & editing:** Miju Son, Youngeun Kim, Donghyo Lee.
